# 60-year-old male with rapidly progressive pneumocephalus caused by Clostridium septicum in the setting of an occult colonic adenocarcinoma

**DOI:** 10.1186/s12879-023-08160-9

**Published:** 2023-03-30

**Authors:** Austin J. Helmink, Taylor A. Wahlig, Paul D. Fey, Jie Chen, Kirk W. Foster

**Affiliations:** Department of Pathology and Microbiology, University of Nebraska Medical Center, Omaha, NE 68198 USA

**Keywords:** *Clostridium septicum*, Pneumocephalus, Colonic adenocarcinoma

## Abstract

**Background:**

Disseminated *Clostridium septicum* infection is an uncommon complication associated with malignancies, particular colonic adenocarcinoma. The organism appears to preferentially colonize large masses in rare individuals and subsequently seed the blood via mucosal ulceration. This has rarely been reported to lead to central nervous system infection and, in several cases, rapidly progressive pneumocephalus. In the few cases reported, this was a universally fatal condition. The current case adds to the reports of this extremely rare complication and provides a unique and complete clinicopathologic characterization with autopsy examination, microscopy, and molecular testing.

**Case Presentation:**

A 60-year-old man with no known past medical history was discovered having seizure-like activity and stroke-like symptoms. Blood cultures turned positive after six hours. Imaging revealed a large, irregular cecal mass as well as 1.4 cm collection of air in the left parietal lobe that progressed to over 7 cm within 8 h. By the following morning, the patient had lost all neurologic reflexes and died. Post-mortem examination revealed brain tissue with multiple grossly evident cystic spaces and intraparenchymal hemorrhage, while microscopic exam showed diffuse hypoxic-ischemic injury and gram-positive rods. *Clostridium septicum* was identified on blood cultures and was confirmed in paraffin embedded tissue from the brain by 16 S ribosomal sequencing and from the colon by *C. septicum* specific PCR.

**Conclusions:**

*C. septicum* is an anaerobic, gram-positive rod that can become invasive and is strongly associated with gastrointestinal pathology including colonic adenocarcinomas. Central nervous system infection with rapidly progressive pneumocephalus is a rarely reported and universally fatal complication of disseminated *C. septicum* infection.

## Introduction

*C. septicum* is an obligate anaerobic, gram-positive rod that produces endospores as well as four major exotoxins (α-, β-, γ- and δ-toxin) that have strong necrotizing properties [1]. Systemic infections with *C. septicum* are strongly associated with malignancy, especially colorectal neoplasms [2]. The organism can seed the blood through mucosal ulceration, and, in rare cases, has been documented to spread to the central nervous system (CNS) and cause rapidly progressive pneumocephalus. Of 21 reported cases of *C. septicum* CNS infection, 13 had pneumocephalus and three had both pneumocephalus and colorectal cancer. These pneumocephalus cases were universally fatal. Here we report an autopsy case of rapidly progressive pneumocephalus secondary to a disseminated *C. septicum* infection in the setting of an occult colonic adenocarcinoma. The diagnosis was confirmed by positive pre-mortem blood cultures as well as brain tissue demonstrating gram positive rods and *C. septicum* positivity on 16s ribosomal sequencing [3]. Overall, this provides a unique clinicopathologic correlation of an extremely rare entity.

## Clinical history

**Fig. 1:**
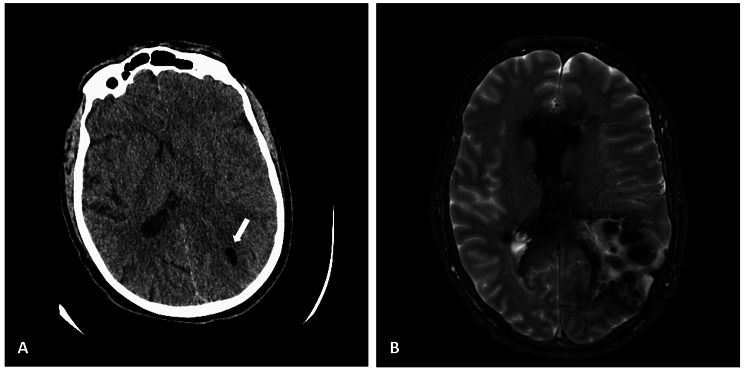
(**A**) CT scan at presentation demonstrating 1.2 cm air collection in left parietal lobe (arrow). (**B**) Brain MRI approximately 8 h later with large (7.3 × 6.1 × 5.3 cm) fluid-gas collection extending into the ventricular system

The patient was 60-year-old man with no known past medical history. The patient reportedly had been lethargic, tired, and not eating well over the prior week. The morning of presentation, a family member heard a noise and discovered the patient arching his back with left gaze deviation, right-sided hemiparesis, and incontinence of bloody stool. In the emergency department he was intubated due to declining respiratory status. Two sets of blood cultures were drawn and turned positive for *Clostridium septicum* after six hours. The patient was started on broad spectrum antibiotics. Computed tomography (CT) head, CT angiography (CTA), and CT perfusion scan were completed per the stroke protocol. Imaging revealed a 1.4 cm collection of air in the left parietal lobe (Fig. 1A). CT abdomen/pelvis was remarkable for a large, irregular cecal mass. Approximately 8 h later, magnetic resonance imaging (MRI) and magnetic resonance venography of the brain showed a large left-sided fluid-gas collection that extended into the lateral ventricle (7.3 × 6.1 × 5.3 cm) (Fig. 1B). By the following morning, the patient had lost all neurologic reflexes. With permission, the patient was made comfort measures only and died that afternoon, approximately 32 h after presentation. The post-mortem interval between death and autopsy was 66 h and 17 min.

**Fig. 2:**
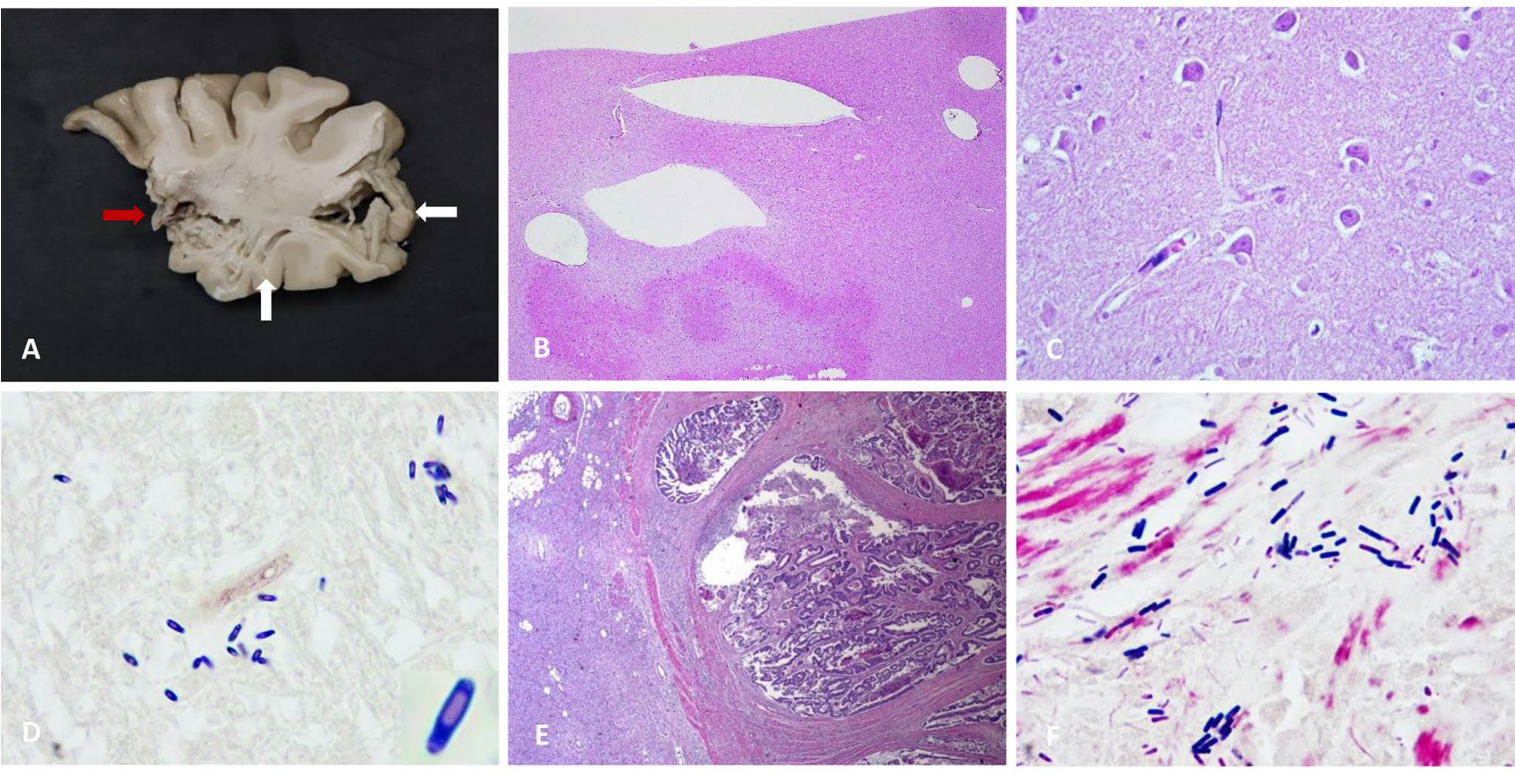
(**A**) Cross section of brain demonstrating architectural disruption with necrosis and intraparenchymal hemorrhage (red arrow) as well as multiple cystic spaces within the cerebellar parenchyma (white arrows). (**B**) Multiple cystic spaces within brain parenchyma (20x). (**C**) Hypoxic-ischemic injury with diffuse hyper-eosinophilic necrotic neurons (400x). (**D**) Gram stain of brain tissue demonstrate gram positive rods with terminal and subterminal spores consistent with C. septicum (1000x; inset shows digital zoom of a single organism). (**E**) Moderately differentiated adenocarcinoma of the cecum with areas of acute inflammation and necrosis (20x). (**F**) Gram stain of necrotic colonic adenocarcinoma demonstrating gram positive rods consistent with C. septicum (1000x)

Post-mortem examination of the patient’s brain revealed diffusely softened and architecturally disrupted cerebral hemispheres with areas of intraparenchymal hemorrhage and multiple cystic spaces (“swiss cheese brain;” Fig. 2A). Microscopic examination showed intraparenchymal hemorrhage and variably sized cystic spaces (Fig. 2B) as well as severe global hypoxic-ischemic injury characterized by extensive eosinophilic neuronal necrosis (Fig. 2C). No significant acute inflammation was noted, but numerous gram-positive spindle-shaped rods with terminal and sub-terminal spores were identified on Gram stain, consistent with *Clostridium* species (Fig. 2D). Gross examination of the deceased’s gastrointestinal system was notable for a large circumferential ulcerated cecal mass with transmural perforation as well multiple small liver nodules (up to 0.4 cm). Gram stains on the liver sections were negative for bacterial organisms. Microscopic examination of the colon mass showed a partially necrotic moderately differentiated adenocarcinoma with invasion into the muscularis propria, as well as transmural acute inflammation with serositis, consistent with the intestinal perforation (Fig. 2E). Gram-positive rods were identified on Gram stain that were morphologically consistent with those seen in the brain (Fig. 2F). The liver nodules were consistent with metastatic adenocarcinoma. The presence of *C. septicum* was confirmed in paraffin embedded tissue by 16 S ribosomal sequencing using NCBI BLAST in the brain and by *C. septicum* specific PCR in the colon [3]. The cause of death was *Clostridium septicum* pneumocephalus secondary to bacteremia, in association with colonic adenocarcinoma.

## Discussion

*Clostridium septicum* is an anaerobic, gram-positive rod that can become invasive and is strongly associated with gastrointestinal pathology including colonic adenocarcinomas [1, 4]. *C. septicum* is hypothesized to preferentially colonize masses of the colon, particularly the cecum, due to its relatively anaerobic and acidic environment which may favor germination of spores [4]. The presence or absence of *C. septicum* as a component of the normal gut microbiome has been difficult to ascertain. Some authors claim the organism is part of the normal colonic flora, but studies have reported the organism’s presence in only 2.8% of humans [5]. *C. septicum* is also known to live in the intestines of herbivores and may be transmitted to humans by a fecal-oral route [5]. Regardless, as the organism colonizes the tumor, it produces a pore-forming cytolytic alpha toxin. [1, 4]. This alpha toxin is structurally and functionally unrelated to the alpha toxin produced by *Clostridium perfringens* [6]. The alpha toxin induces tissue necrosis leading to mucosal ulceration and subsequent hematogenous spread to distant sites. Thus, opportunistic colonization by *C. septicum* of a large cecal adenocarcinoma with a highly acidic and anaerobic microenvironment followed by organism germination, mucosal ulceration, and vascular invasion provides a plausible mechanism for the pathogenesis of invasive *C. septicum* infections.

In the current case, *C. septicum* spread to the blood and subsequently to the brain, as evidenced by blood cultures positive for *C. septicum*, gram-positive organisms consistent with *C. septicum* in brain tissue, and *C. septicum* detection by 16s ribosomal sequencing on brain paraffin sections. Due to the presence in the colon of bacteria morphologically similar to those in the central nervous system, the colon is favored as being the nidus for sepsis in this patient; however, it is also possible the liver could have acted as the source, particularly due to the presence of metastatic adenocarcinoma. Central nervous system infection then led to rapidly progressive pneumocephalus, a rarely reported complication of disseminated *C. septicum* infection [1, 5, 7]. A 2016 case report by Macha et al. described 19 published reports of *C. septicum* central nervous system infection [1]. Our own literature review discovered an additional two cases published since 2016, [5, 7] for a total of 21 reported cases of *C. septicum* CNS infection. In total, 13 of these cases reported pneumocephalus with at least three having concurrent colorectal carcinoma [1]. Moreover, of the five pneumocephalus cases that included brain tissue microscopic examination, three reported a significant lack of inflammatory infiltrate, a finding that was also noted in our case. This paucity of inflammation has also been noted in cases of *C. septicum* myonecrosis [4]. One hypothesis is that the alpha toxin is able to induce selective apoptosis of neutrophils, which promotes organism growth and may also play a role in tumor progression in cases associated with colorectal carcinoma [4].

Rapid recognition and treatment is vital in order to have any chance of patient survival. Diagnosis rests on recognizing potential sepsis in the setting of colon cancer, having a high index of suspicion for infection with *C. septicum* and understanding its rapidly progressive nature, and initiating aggressive treatment and close neurological observation while awaiting growth of the anaerobic organism in blood cultures. Unfortunately, while several attempts have been made to develop an assay for the organism’s alpha toxin in the setting of animal infections [3, 8], no clinical assay is available for *C. septicum* detection in humans. Overall mortality in the aforementioned 21 cases was 76% (16/21) while mortality in the cases with pneumocephalus was 100% (13/13).^1^ Moreover, most patients with pneumocephalus died within 48 h (85%, 11/13). Our patient was found to have rapidly progressive pneumocephalus causing severe global hypoxic injury within 24 h and death within 36 h of presentation. The mechanism of blood-brain-barrier penetration by *C. septicum* is unclear and may simply be related to vascular damage secondary to sepsis; however, it is worth noting that on further review by us, six of the previously noted 21 cases reported varying degrees of intraparenchymal hemorrhage/hemorrhagic stroke, which was also noted in our case. A plausible mechanism for CNS penetration by *C. septicum* could be microvascular damage due to a combination of severe sepsis and alpha toxin production resulting in intracerebral hemorrhage and organism dissemination within the brain parenchyma.

## Data Availability

No data sets were generated for this case report.
